# Chitosan-Based Bio-Composite Modified with Thiocarbamate Moiety for Decontamination of Cations from the Aqueous Media

**DOI:** 10.3390/molecules25010226

**Published:** 2020-01-06

**Authors:** Nisar Ali, Adnan Khan, Muhammad Bilal, Sumeet Malik, Syed Badshah, Hafiz M. N. Iqbal

**Affiliations:** 1 Key Laboratory for Palygorskite Science and Applied Technology of Jiangsu Province, National & Local Joint Engineering Research Center for Deep Utilization Technology of Rock-salt Resource, Faculty of Chemical Engineering, Huaiyin Institute of Technology, Huaian 223003, China; 2 Institute of Chemistry, University of Campinas, UNICAMP, P.O. Box 6154, Campinas 13084-971, SP, Brazil; 3 Institute of Chemical Sciences, University of Peshawar, Khyber Pakhtunkhwa 25120, Pakistan; adnankhan@uop.edu.pk (A.K.); sumeetmalik1995@gmail.com (S.M.); 4 School of Life Science and Food Engineering, Huaiyin Institute of Technology, Huaian 223003, China; bilaluaf@hotmail.com; 5 Department of Chemistry, Gomal University, D. I. Khan, Khyber Pakhtunkhwa 25120, Pakistan; syedbadshah_hej@yahoo.com; 6 Tecnologico de Monterrey, School of Engineering and Sciences, Campus Monterrey, Ave. Eugenio Garza Sada 2501, Monterrey CP 64849, Mexico

**Keywords:** chitosan, acrylonitrile, bio-composite, Langmuir model, decontamination

## Abstract

Herein, we report the development of chitosan (CH)-based bio-composite modified with acrylonitrile (AN) in the presence of carbon disulfide. The current work aimed to increase the Lewis basic centers on the polymeric backbone using single-step three-components (chitosan, carbon disulfide, and acrylonitrile) reaction. For a said purpose, the thiocarbamate moiety was attached to the pendant functional amine (NH_2_) of chitosan. Both the pristine CH and modified CH-AN bio-composites were first characterized using numerous analytical and imaging techniques, including ^13^C-NMR (solid-form), Fourier-transform infrared spectroscopy (FTIR), elemental investigation, thermogravimetric analysis, and scanning electron microscopy (SEM). Finally, the modified bio-composite (CH-AN) was deployed for the decontamination of cations from the aqueous media. The sorption ability of the CH-AN bio-composite was evaluated by applying it to lead and copper-containing aqueous solution. The chitosan-based CH-AN bio-composite exhibited greater sorption capacity for lead (2.54 mmol g^−1^) and copper (2.02 mmol g^−1^) than precursor chitosan from aqueous solution based on Langmuir sorption isotherm. The experimental findings fitted better to Langmuir model than Temkin and Freundlich isotherms using linear regression method. Different linearization of Langmuir model showed different error functions and isothermal parameters. The nonlinear regression analysis showed lower values of error functions as compared with linear regression analysis. The chitosan with thiocarbamate group is an outstanding material for the decontamination of toxic elements from the aqueous environment.

## 1. Introduction

Fast industrialization, developing urbanization, expanding populace, broad utilization of nonmaintainable assets, and uncontrolled mishandling of common resources are making destructive, unrecoverable, and grave harms to the earth [1]. These effluents are persistent in the ecosystem and difficult to decontaminate [2]. Among these pollutants, one of the most significant is the heavy metals. Fast worldwide development in the passage of the last century has raised the extent of heavy metals in surface and groundwater through releases of wastewater generated from metallurgical, mining, and substance- and battery-producing businesses [3,4]. The most substantial metals, copper and lead, are amazingly poisonous, genuinely affecting all types of life, including human beings, when exceeding their permitted levels [5,6]. Heavy metals are likewise not biodegradable and can accumulate in living beings [7,8]. The release of these metals is lethal and perceived as mutagenic, cancer causing, or teratogenic [9,10,11].

The utilization of such unhygienic water prompts health risks and can harm the human organs, for example, the kidney, liver, cerebrum, focal apprehensive system, and so on, or, on the other hand, can be susceptible to the skin [12,13]. Among these metals, copper and lead at high fixations can be destructive to a biological system and creatures and cause numerous human illnesses, like sickness, frailty, and pancreas harm. Copper and lead discover applications in electroplating, paint, metal completing, and mining ventures. As indicated by the World Health Organization, the permitted uptake for copper in water is 0.05 mg/L. The allowable lead quantity in water uptake is 0.010 ppm. Therefore, different organic and physicochemical techniques, for example, ion exchange [14], chemical coagulation [15], flocculation [16], precipitation/flotation [17], microbial [18], photocatalysis [19], and sorption [20,21], have been utilized in the treatment of wastewater. Ion exchange is associated with economic constraints (initial cost of the selective resin, maintenance costs, regeneration time-consuming, etc.), and large volume requires large columns, rapid saturation, and clogging of the reactors. Precipitation, coagulation, and flocculation require high chemical consumption, high sludge production, handling, and disposal problems. Initial investment costs, transport, and storage of the effluents and formation of secondary pollutants are some of the disadvantages of the photocatalysis process. Microbial treatment requires management and maintenance of the microorganisms and/or physicochemical pretreatment to prevent the production of toxic metabolites by microbes [22,23]. Sorption is considered as a promising methodology because of its minimal effort, adequacy, and no auxiliary contamination [24]. Subsequently, picking fitting materials as sorbents can adequately mitigate water contamination by natural colors. As of late, materials acquired from sustainable assets, for example, chitosan, sodium alginate, cellulose, and starch [25], are rising as a potential elective sorbent for metal ions from effluents.

A biopolymer named chitosan is acquired through the chitin *N*-deacetylation, which is another abundant biopolymer in nature, after cellulose, and supporting corporeal of shellfish, insects, and so forth. Moreover, chitosan exhibits natural and chemical effects, including nonpoisonous quality, biocompatibility, heightened reactivity, chirality, antibacterial ability, chelation, and sorption effects [26]. Chitosan is entrenched as a superb regular sorbent, as its hydroxyl (OH) as well as amine (NH_2_) act as strategic sites forming complexes with different cations. The amino and hydroxyl groups likewise permit the physical arrangement (film, globules, and nanoparticles) and chemical alteration of biopolymer chitosan. The chemical reaction with chitosan is normally done to attach the Lewis basic centers on chitosan polymeric backbone for higher take-up of metal ions [27,28]. Because of the poor sorption performance and stability of pristine chitosan, there is need for anchoring of Lewis basic centers to chitosan using a facile and low-cost way of preparation [29].

To improve the sorption of chitosan towards metal ions, the chemical alteration of chitosan in a one-step three-parts reaction has been done [30]. The chitosan, acrylonitrile, and carbon disulfide were utilized as reactants to embed thiocarbamate moiety in the polymeric chain. ^13^C-NMR in solid state, FTIR spectroscopy, thermogravimetric investigation, scanning electron microscopy, and elemental analysis were utilized to elucidate the chitosan alteration. The novel material was utilized for the decontaminating of copper and lead from aqueous medium. Langmuir model was utilized to assess the greatest sorption limit of the altered material for these cations. Other isothermal models applied to sorption study were Temkin and Freundlich isotherms, both in straight/nonlinear regression analysis. Langmuir model in different linearized structures was used for investigative information to match parameters of the isotherms.

## 2. Results and Discussion

### 2.1. Elemental Analysis

The anchoring of thiocarbamate moiety to the chitosan (CH) pendant chain was confirmed by determining the sulfur, carbon, and nitrogen content of the modified chitosan. The percentage, quantity L_o_ (mmol g^−1^), and C/N of these elements were determined using Equation (1) [31], as shown in Table 1. The presence of sulfur contents (1.24 mmol g^−1^) based on the sulfur elemental analysis in chemically modified chitosan showed the addition of thiocarbamate moiety in the pristine chitosan. Also, the change in the amount of nitrogen (5.14 mmol g^−1^) and carbon (31.79 mmol g^−1^) in the altered chitosan, as well as change in C/N ratio, confirm the modification, as shown in Table 1.

(1) $$L_{0}=\frac{\%\;Element\times 10}{Atomic\;mass\;of\;element}$$

### 2.2. Infrared Spectroscopy

**Figure 1:**
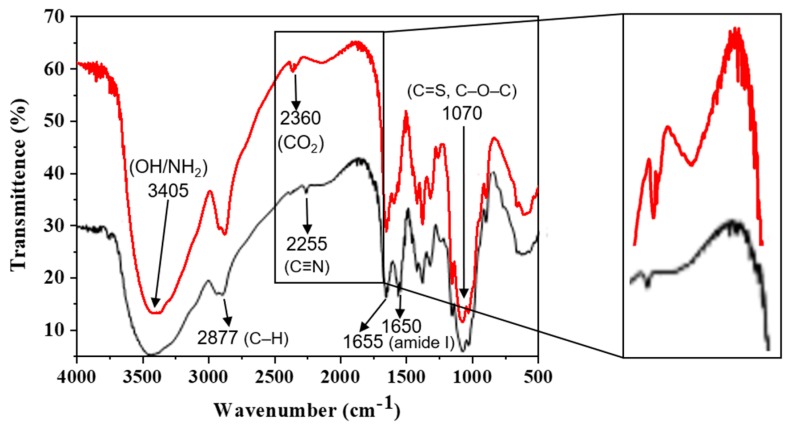
Infrared spectra of CH-based bio-composites, that is, pristine CH bio-composite (red line) and chemically modified CH-AN bio-composite (black line), along with zoomed area showing modification.

The FTIR spectroscopy was used to confirm the addition of thiocarbamate moiety in the chitosan by comparing the spectrum of pristine chitosan with altered chitosan, as seen from Figure 1. The chitosan FTIR spectrum portrayed distinctive wide bands at 3405 cm^−1^ due to OH/NH_2_. The stretching vibrations for C–H were turned out at 2916 and 2877 cm^−1^. The band associated with amide I was present at 1655 cm^−1^. The N–H and primal alcohol bond showed bands at 1592 cm^−1^ and 1426 cm^−1^. The CH_3_ symmetrical deformation was seen at 1419 cm^−1^. The stretching vibrations for the C–O bond had emerged at 1076 cm^−1^ of the β-glycosidic bond amongst carbon 1 and 4 [32]. The chemically altered chitosan spectra (CH-AN) with thiocarbamate showed the same bands along with appearance of a new band at 2255 cm^−1^, which is accredited to C≡N bond [33]. The band appeared at 1550 cm^−1^ corresponds to N–H deformation. The band at 1076 cm^−1^ due to C–O bond turned highly intense and shifted to a smaller value of 1072 cm^−1^, which is identical to C=S stretching vibrations [24,27].

### 2.3. Nuclear Magnetic Resonance

**Figure 2:**
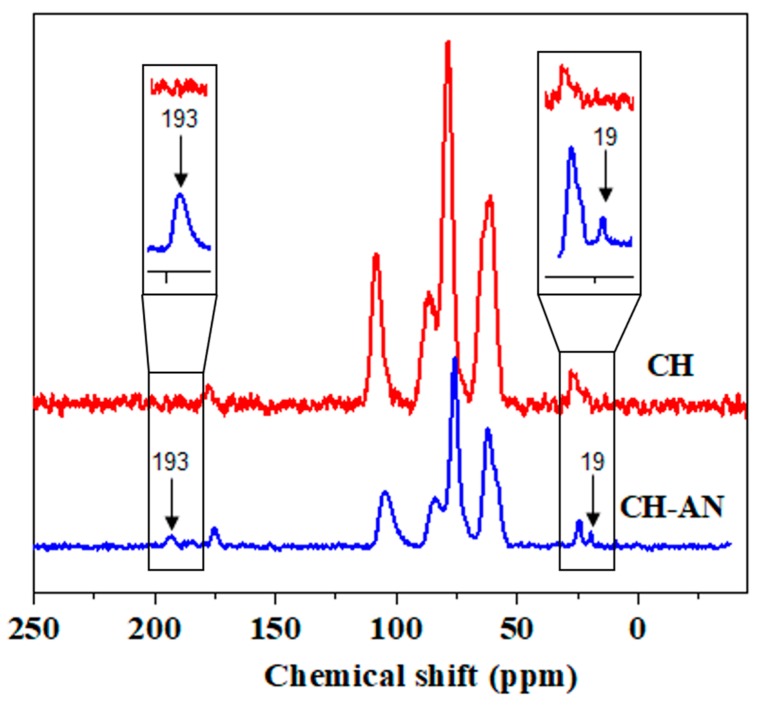
Carbon NMR spectra in solid state of CH-based bio-composites, that is, pristine CH bio-composite (red line) and chemically modified CH-AN bio-composite (blue line).

The ^13^C nuclear magnetic resonance spectrum for pristine chitosan in solid form (CH) showed five distinctive peaks. The peak at 75 ppm corresponds to C3/C5 of the chitosan pendant chain. The peaks for C6, C4, C2, and C1 of the chitosan chain have appeared at 62, 84, 58, and 105 ppm. The peak of an acetylated methyl group and carbonyl of chitin appeared at 24 and 175 ppm, as shown in Figure 2 [34]. As presumed, the chemically altered chitosan (CH-AN) gave new peaks along with chitosan characteristic peaks. The chitosan (CH-AN) showed a peak at 193 ppm, which is due to C=S double bond of thiocarbamate. A peak at 19 ppm is related to CH_2_ carbon of the attached thiocarbamate moiety, as shown in Figure 2.

### 2.4. Thermogravimetry

**Figure 3:**
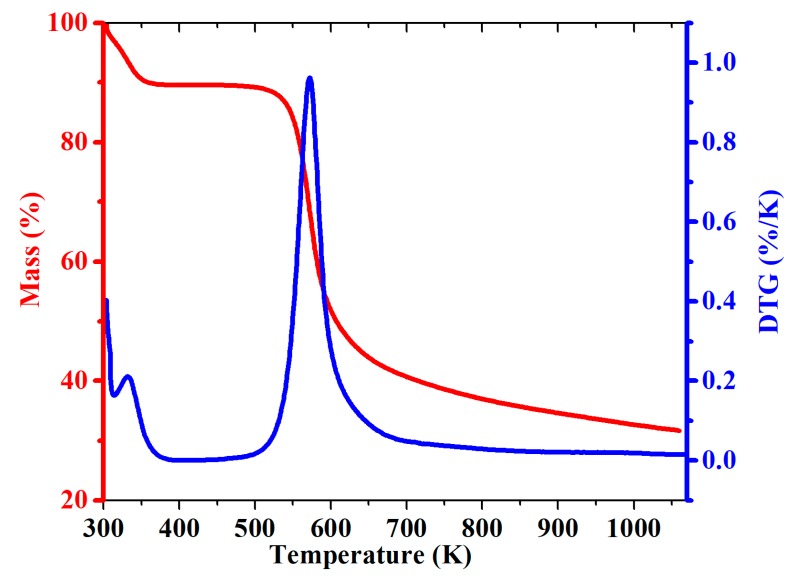
Thermogravcimetric (red line) and derivative curves (blue line) for pristine CH biopolymer. Thermogravcimetric and derivative curves for pristine CH biopolymer.

**Figure 4:**
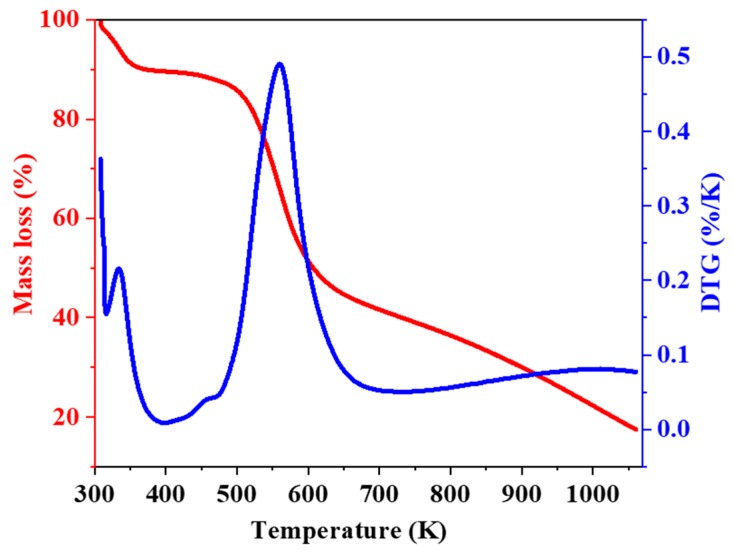
Thermogravcimetric (red line) and derivative curves (blue line) for chemically modified CH-AN bio-composite.

The chitosan (CH) and modified material (CH-AN) showed different thermal degradation profiles, as shown in Figure 3 and Figure 4. The derivative curves showed two clear peaks for chitosan (CH) and modified chitosan (CH-AN), confirming that two thermal events are involved in the decomposition process. The first step at which loss of mass of chitosan occurred appeared in 326–350 K, which is because of the water loss physically sorbed on chitosan or linked through hydrogen bonding. As can be seen from the derivative curve in Figure 3, the maximum loss of mass (9%) occurred at 333 K.

The degradation of biopolymer occurs at 551–601 K with mass loss of 57%, and maximum peak appeared at 570 K [31]. At this temperature, the glycosidic bonds of polysaccharides start random division to butyric acid, acetic acid, and other lower-molecular-weight products [31]. The chemically modified chitosan also presented two thermal events one with the release of water at 335 K with 9% of mass loss. The following step of thermal degradation for synthetically altered chitosan occurred at lowered temperature, and a greater peak appeared at 559 K with a mass loss of 50%, obtained from integral curve. This shows that chemically modified chitosan is less stable than pristine chitosan, which may be due to the rupturing of intra and intermolecular hydrogen bonds in the polymeric chain of chitosan structure with insertion of a new molecule.

### 2.5. Scanning Electron Microscopy

**Figure 5:**
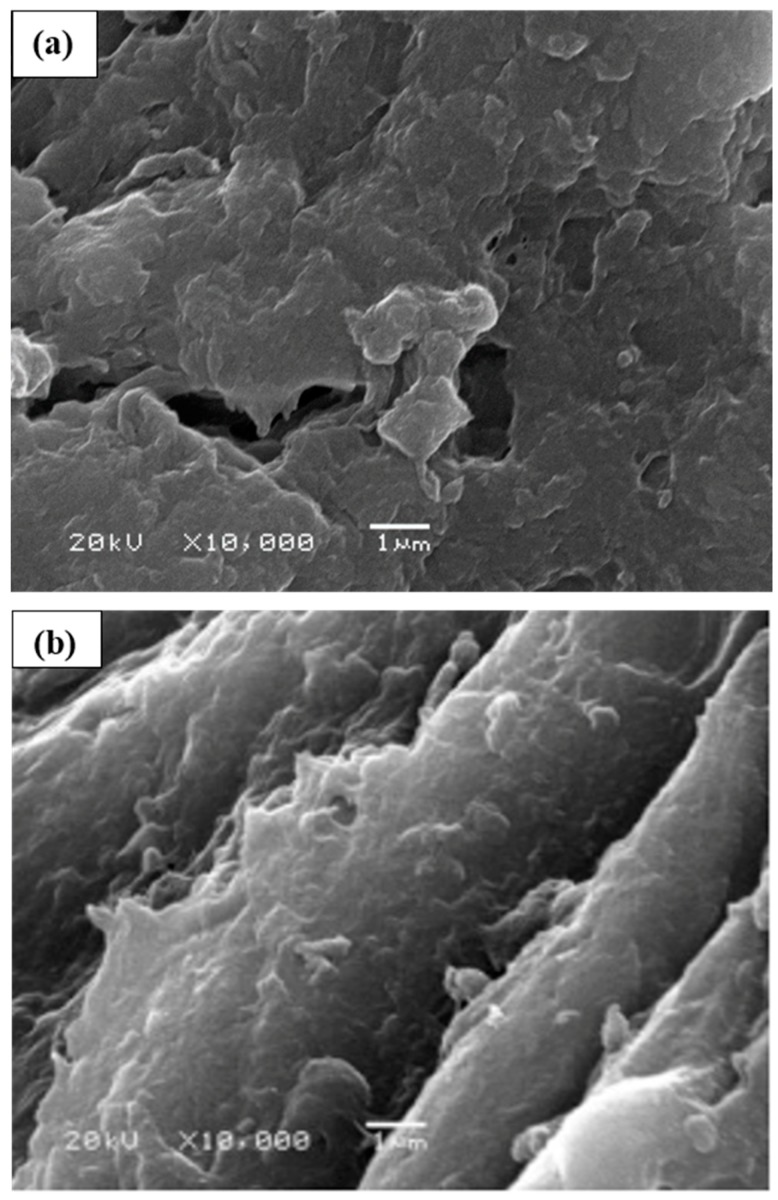
SEM images of CH-based bio-composites, that is, pristine CH bio-composite (**a**) and chemically modified CH-AN bio-composite (**b**).

The surface morphology and topography of the chitosan (CH) and synthetically altered chitosan (CH-AN) had been obtained from images of scanning electron microscopy, as can be seen in Figure 5. The pristine chitosan portrayed a compact structure, as shown in Figure 5a. The smooth and stiff surface of chitosan is due to the intra and intermolecular hydrogen bond framework involved with every single polymeric chain to keep up their firmness [31]. While the chemically altered chitosan (CH-AN) showed a surface that has clear channels along the surface of the biopolymer, as indicated in Figure 5b. The insertion of an external molecule can upset this arrangement of bonds in a reaction. Hence, the CH-AN has rough and scramble-like surface, providing easy site availability to cations present in aqueous medium.

### 2.6. Sorption Studies

The Lewis basic centers sulfur, nitrogen, and oxygen in contact with pristine and CH-AN have been used for the sorption of copper and lead from aqueous solutions. The quantity of these divalent cations sorbed (*Nf)* by the biopolymers was obtained through Langmuir sorption isotherm, as can be seen in Table 2. The Langmuir constant, the interaction energy (*b*), maximum sorption capacity (*Ns*), and correlation coefficient (r) were determined from the Langmuir model linear form, as shown in Table 2. The synthetically altered chitosan portrayed improved sorption for copper and lead compared with precursor chitosan due to the insertion of new sites for metal sorption. The sorption of Pb^2+^ and Cu^2+^ on modified chitosan (CH-AN) was higher than that of precursor chitosan (Pb^2+^ 1.40 mmol g^−1^, Cu^2+^ 1.19 mmol g^−1^), as shown in Table 2. The chitosan (CH-AN) contains thiocarbamate moiety that has sulfur Lewis basic centers, which has a higher affinity for lead [24,27]. The ion radius of Pb^2+^ ion is higher than that of Cu^2+^ ion. Pearson’s hard and soft acids and bases (HSAB) theory described that sulfur acts as a soft base, bound with a large and highly polarizable Pb^2+^ ion. Lead ion is softer and more polarizable than copper ion, forming a strong bond with sulfur due to soft–soft interaction [35].

The affinity of sorbents with sorbate, surface properties and mechanism of sorption, is well understood from the parameters of isothermal equations. The most common isotherms the Langmuir, Freundlich, and Temkin models have been practiced in the sorption process. The Langmuir model in four different linear forms, in both linear and nonlinear regression analysis has been practiced for the sorption procedure to yield better results. These different models showed different values of correlation coefficients and constants, as shown in Table 2 [36]. The greater correlation coefficient value, minimum error functions chi-square, χ^2^, and SE values confirm that the Langmuir isotherm fits best to experimental data suggesting monolayer sorption on the surface. Also, the sorbate–sorbate interaction is imperceptible, and every molecule shows similar activation energy. The Freundlich and Temkin models portrayed higher values of error functions than the Langmuir model, as can be depicted in Table 3, and were least befitting to describe the sorption process. The nonlinear regression analysis of the three models showed different values of constant, as shown in Table 4.

These models were also applied to data using nonlinear regression analysis to retrieve the isothermal parameters. The parameters obtained from this method for Langmuir were close to those from its linearized form. However, error functions were smaller than in a linearized form of Langmuir model. The error functions of the nonlinear form of Langmuir model were also lower than those of the Freundlich and Temkin models, as depicted in Table 4, for synthetically altered chitosan (CH-AN). The perfect way for finding the isotherm fitting to experimental data is the nonlinear approach that reduces the dissemination of error amongst analytical findings and isotherm used [31].

## 3. Materials and Methods

### 3.1. Chemicals, Reagents, and Materials

Deacetylated chitosan (78%) was obtained from Primex Ingredients A.S. (Haugesund, Norway). Analytical-grade and purified products including acrylonitrile (Aldrich, St. Louis, MO, USA), carbon disulfide, ethanol, triethylamine (Synth), lead, and copper nitrates (Vetec) were used.

### 3.2. Synthesis and Modification of CH Bio-Composite

**Figure 6:**
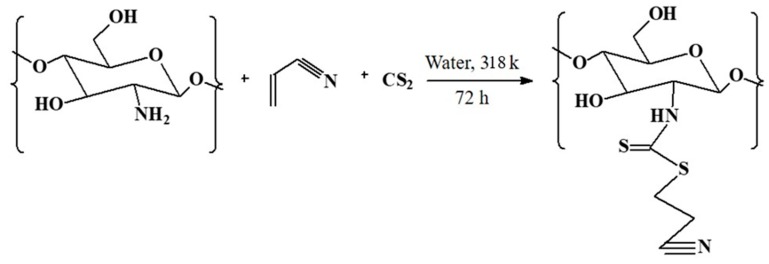
The proposed reaction mechanism for chitosan modification using acrylonitrile (AN) in the presence of carbon disulfide. Triethylamine was additionally supplemented to the suspension as a catalyst.

The modification of chitosan was performed with thiocarbamate moiety through Michael’s addition reaction. The reaction was carried out in an individual-step, three-components reaction using acrylonitrile as a Michael acceptor. Following a common pathway, a mixture of chitosan (3.0 g) and carbon disulfide (1.80 cm^3^) was suspended in a 200 cm^3^ of water. Triethylamine (0.5 cm^3^) was added to this suspension as a catalyst at 318 K. Then, 0.80 cm^3^ of acrylonitrile was added to this mixture. The suspension stirring was done mechanically for 72 h at 318 K in nitrogen-protected environment. The chitosan (CH-AN) was separated further through filtration, followed by washing with ethanol and water. The material was dehydrated provided 323 K per 6 h under vacuum, as shown in Figure 6.

### 3.3. Characterization of CH-Based Bio-Composites

The sulfur, carbon, and nitrogen content of the chitosan and modified chitosan was found out using the elemental analyzer of Perkin Elmer model PE 2400. In order to find out the FTIR spectra, sample with KBr pellets was used with an IR spectra Bomem Spectrophotometer, MB-series, with a resolution ranging from 4000–400 cm^−1^ with the help of Fourier transformation, resulting in 32 scans. For the ^13^C-NMR spectra in solid state, a Bruker AC 300/P spectrometer was used applying CP/MAS technique. At 75.47 MHz frequencies, calculations were performed alongside 4 kHz angle spinning, 5 s pulse revival, and 1 ms contact times. Thermogravimetric curves were obtained in an argon atmosphere at a flow rate of 30 cm^3^ s^−1^, using Shimadzu TGA 50 apparatus providing 0.167 K s^−1^ heat rate. The concentration of cations in equilibrium was measured by a PE 3000 DV ICP-OES.

### 3.4. Sorption Potential of CH-AN Bio-Composite

The thiocarbamate chitosan (CH-AN) capacity for the sorption of cations was evaluated using the batch process in a duplicate run. A polyethylene bottle was taken containing 20 mg of the product. Then, metal solution (25.0 cm^3^ of metal nitrate solution) was added to these flasks in concentration (7.0 × 10^−4^ to 2.0 × 10^−3^ mol dm^−3^) and was shaken for 24 h at 298 ± 1 K to achieve isothermic superabundance. The time required by the cations to acquire isothermal saturation was similar to cations solution previously used to carry out kinetic experiments and obtained after 4 h of contact time. The supernatant solution and sorbent were separated through decantation to determine the number of cations sorbed on the material. The ICP-OES was used to find out the number of cations in the sample before and after sorption. The number of cations retained by the thiocarbamate chitosan (CH-AN) was determined (mmol g^−1^) using Equation (2). In Equation (2), the number of cations is represented by Nf, ni (the moles no. of cations in the initial solution), ns (supernatant at equilibrium), and utilized sorbent’s mass in the experiment, is shown, by m [37].

(2) $$Nf=\frac{ni-ns}{m}$$

Four different types of Langmuir models as well as Temkin and Freundlich models with linear/nonlinear approaches have been carried out for the experimental data.

### 3.5. Isotherm Models

The interaction between sorbent and sorbate molecule is explained well using isothermal models. The ability of the modified chitosan to sorb metal ions is determined by applying these models. The number of cations sorbed and its relationship with a remaining concentration in solution is better described by isothermal models, as shown in Table 5 [30,36,38]. The sorption process was exercised with the Langmuir, Freundlich, and Temkin isotherms applying linear and nonlinear regression methods, as shown in Table 5.

#### 3.5.1. Langmuir Isotherm

The Langmuir isotherm can be linearized into four forms, which were used for sorption results as shown in Table 5. Where the term *Nf* (mmol g^−1^) represents the number of cations retained on the sorbent and *Cs* (mmol dm^−3^) shows an assemblage of sorbate at equilibrium, respectively. The maximum sorption capacity is denoted by *Ns* (mmol g^−1^), and *b* (dm^3^ mmol^−1^) is Langmuir constant. The equilibrium constant shows the affinity of sorbents towards sorbate [30].

#### 3.5.2. Freundlich Isotherm

The surface’s heterogeneity, active site energy, and exponential distribution are covered by the Freundlich isotherm. This isotherm points out multiple layered sorptions at sorbent [38]. The Freundlich model, in both nonlinear and linear forms is shown in Table 5. In Freundlich equation, *Nf* is the measure of cations sorbed at equilibrium (mmol g^−1^) and Cs is the cations concentration at equilibrium in supernatant (mmol dm^−3^). The Freundlich constant and capacity factor are represented by K_F_ and n [31].

#### 3.5.3. Temkin Isotherm

Heat capacity of sorption shows a linear decrease when the sorbate surface is covered with sorbents due to interaction between them, described by a uniform distribution of binding energies. Both the linear and nonlinear forms of the Temkin isotherms are presented in Table 5. The Temkin constants *K_T_*, *n_T_*, and *b* are obtained from the plot of ln *Cs* versus *Nf*. The sorption heat (*b*) is found out through Equation (3).

(3) $$n_{T}=\frac{RT}{b}$$

The optimization procedure is enabled by an error function, which better describes the isothermal model fitting to experimental data. The match of isothermal models to analytical findings is determined based on chi-square, χ^2^, the standard error (SE), and correlation coefficient (R), as can be seen from Table 5. Where *Nf*_exp_ is the moles no. sorbed, *Nf*_calc_ is the no. of moles found out for every model, *m* is the no. of points in every isotherm, and *P* is the no of parameters in every equation [39].

## 4. Conclusions

The pendant chain of chitosan was successfully anchored with the thiocarbamate group to add Lewis basic centers and improve its sorption capacity towards lead and copper. The attachment of thiocarbamate moiety was confirmed using infrared absorption and nuclear magnetic resonance spectroscopy in the solid form, elemental findings, and scanning electron microscopy. The altered material portrayed greater sorption ability from aqueous solution of lead and copper based on Langmuir sorption isotherm than pristine chitosan. The Langmuir, Freundlich, and Temkin models were applied to experimental data using both linear and nonlinear regression analysis. The linearized form Langmuir fits better with experimental data than Freundlich and Temkin’s isothermal models considering correlation coefficient and error functions. The Langmuir model in four different forms gave different isothermal parameters and values of error functions, which may be due to transformation of nonlinear form into linear equations. The accuracy of the results is influenced by the distribution of error due to different arrangement of axes that leads to negligence of theory behind the isotherm. While applying the nonlinear methodology, least error values were obtained for the three models without any problems.

## Figures and Tables

**Table 1:** Percentages of carbon (C) and nitrogen (N), number of moles for chitosan (CH) and chemically modified chitosan (CH-AN), and the corresponding molar ratio (C/N).

Sample	C/%	N/%	S/%	C/mmolg^−1^	N/mmolg^−1^	S/mmolg^−1^	C/N
CH	40.63	7.39	−	33.86	5.27	−	6.42
CH-AN	38.15	7.22	3.99	31.79	5.14	1.24	6.18

**Table 2:** Number of moles sorbed (*Nf*), parameters of the Langmuir (*Ns* and *b*), correlation coefficients (r), and standard errors (SE) for the interaction of divalent metals with modified chitosan CH-AN at 298 ± 1 K using linear method.

Isotherm	Constant	Type I	Type II	Type III	Type IV
Cu^2+^	*Nf* (mmol g^−1^)	2.02	2.02	2.02	2.02
	*N*s (mmol g^−1^)	2.83 ± 0.02	2.82 ± 0.01	2.83 ± 0.02	2.84 ± 0.01
	*b* (g mmol^−1^)	0.30 ± 0.01	0.30 ± 0.01	0.30 ± 0.05	0.30 ± 0.01
	R^2^	0.998	0.999	0.997	0.997
	SE	0.019	0.019	0.019	0.019
	χ^2^	0.001	0.001	0.001	0.001
Pb^2+^	*Nf* (mmol g^−1^)	2.54	2.54	2.54	2.54
	*N*s (mmol g^−1^)	2.99 ± 0.03	2.99 ± 0.03	2.99 ± 0.03	2.99 ± 0.03
	*b* (g mmol^−1^)	0.31 ± 0.01	0.31 ± 0.01	0.31 ± 0.01	0.31 ± 0.01
	R^2^	0.998	0.998	0.998	0.998
	SE	0.032	0.032	0.032	0.032
	χ^2^	0.005	0.005	0.005	0.005

**Table 3:** Number of moles sorbed (*Nf*), parameters of the Freundlich (*n* and *Kf*), Temkin (*b* and *K_T_*), correlation coefficients (*r*), and respective error for the interaction of divalent metals with chitosan CH-AN at 298 ± 1 K, using a linear method.

Isotherm	Constant	Cu(II)	Pb(II)
Freundlich	*K_f_* (mmol g^−1^)	0.61 ± 0.01	0.91 ± 0.02
	*n*	1.61 ± 0.03	2.56 ± 0.02
	R^2^	0.975	0.954
	SE	0.21	0.42
	χ^2^	3.55	0.928
Temkin	*K_T_* (mmol dm^−3^)	3.55 ± 0.03	2.96 ± 0.04
	*b* (kJ mol^−1^)	1.46 ± 0.02	1.64 ± 0.02
	R^2^	0.987	0.984
	SE	0.03	0.05
	χ^2^	0.09	0.021

**Table 4:** Number of moles sorbed (*Nf*), parameters of the Freundlich (*n* and *K_f_*), Temkin (*K_T_* and *b*), correlation coefficients (*r*), and respective error for the interaction of Cu^2+^ and Pb^2+^ with chitosan, CH-AN at 298 ± 1 K, using a nonlinear method.

Isotherm	Constant	Cu(II)	Pb(II)
Langmuir	*Nf* (mmol g^−1^)	2.02	2.54
	*N*s (mmol g^−1^)	2.87 ± 0.03	3.02 ± 0.03
	*b* (g mmol^−1^)	0.29 ± 0.01	0.30 ± 0.01
	R^2^	0.999	0.996
	χ^2^	0.002	0.001
Freundlich	*K_f_* (mmol g^−1^)	0.70 ± 0.04	0.98 ± 0.06
	*n*	1.89 ± 0.12	2.87 ± 0.22
	R^2^	0.982	0.948
	χ^2^	0.007	0.017
Temkin	*K_T_* (mmol dm^−3^)	3.52 ± 0.32	2.96 ± 0.32
	*b* (kJ mol^−1^)	1.68 ± 0.06	1.50 ± 0.05
	R^2^	0.988	0.986
	χ^2^	0.005	0.004

**Table 5:** Sorption isotherm models, the linear and nonlinear representations, and respective plot graphical forms.

Isotherm	Nonlinear Form	Linear Form	Plot
Langmuir type 1	$Nf=\frac{NsbCs}{1+bCs}$	$\frac{Cs}{Nf}=\frac{Cs}{Ns}+\frac{1}{Nsb}$	$\frac{Cs}{Nf}\;vs\;Cs$
Langmuir type 2	−	$\frac{1}{Nf}=\left[\frac{1}{Nsb}\right]\frac{1}{Cs}+\frac{1}{Ns}$	$\frac{1}{Nf}\;vs\;\frac{1}{Cs}$
Langmuir type 3		$Nf=Ns-\left[\frac{1}{b}\right]+\frac{Nf}{Cs}$	$Nf\;vs\;\frac{Nf}{Cs}$
Langmuir type 4		$\frac{Nf}{Cs}=bNs-bNf$	$\frac{Nf}{Cs}\;vs\;Nf$
Freundlich	$Nf=KfCs^{\frac{1}{n}}$	$\log Nf-\log Kf+\frac{1}{n}logCs$	logCs vs logNf
Temkin	$Nf=\ln{\left(K_{T}Cs\right)}^{\frac{1}{n_{T}}}$	$Nf=n_{T}\ln K_{T}+n_{T}\ln Cs$	$Nf=n_{T}\ln K_{T}+n_{T}\ln C$
Standard Error	$SE=\sqrt{\frac{1}{m-P}}\sum_{i=1}^{m}{\left({Nf}_{\exp i}-{Nf}_{cali}\right)}^{2}$		
Chi-square	$x^{2}=\sum_{i=1}\frac{{\left({Nf}_{\exp i}-{Nf}_{cali}\right)}^{2}}{{Nf}_{cali}}$		
